# Potentiality of Antibacterial Gels for the Prophylactic Coating of Hernia Repair Prosthetic Materials

**DOI:** 10.3390/gels10110687

**Published:** 2024-10-24

**Authors:** Bárbara Pérez-Köhler, Selma Benito-Martínez, Celia Rivas-Santos, Verónica Gómez-Gil, Francisca García-Moreno, Gemma Pascual

**Affiliations:** 1 Departamento de Medicina y Especialidades Médicas, Facultad de Medicina y Ciencias de la Salud, Universidad de Alcalá, 28805 Alcalá de Henares, Spain; barbara.perez@uah.es (B.P.-K.); selma.benito@uah.es (S.B.-M.); celia.rivas@uah.es (C.R.-S.); 2 Biomedical Research Networking Center in Bioengineering, Biomaterials and Nanomedicine (CIBER-BBN), 28029 Madrid, Spain; veronica.gomezg@uah.es (V.G.-G.); francisca.garciamorenonisa@gmail.com (F.G.-M.); 3 Ramón y Cajal Health Research Institute (IRYCIS), 28034 Madrid, Spain; 4 Departamento de Ciencias Biomédicas, Facultad de Medicina y Ciencias de la Salud, Universidad de Alcalá, 28805 Alcalá de Henares, Spain

**Keywords:** antibacterial coating, carboxymethylcellulose, chlorhexidine, hernia repair, infection prevention, mesh, MRSA, polypropylene, rifampicin, *Staphylococcus aureus*

## Abstract

Prosthetic mesh infection constitutes one of the major postsurgical complications following abdominal hernia repair. Antibacterial coatings represent a prophylactic strategy to reduce the risk of infection. This study assessed the in vitro response of two antibacterial gels made of 1% carboxymethylcellulose (CMC) functionalized with an antiseptic (chlorhexidine, CHX) or an antibiotic (rifampicin, RIF), developed for the coating of polypropylene (PP) meshes for hernia repair. Fragments of a lightweight PP mesh (1 cm^2^) presoaked in the unloaded or drug-loaded CMC (0.05% CHX; 0.13 mg/mL RIF) were challenged with 10^6^ CFU/mL *Staphylococcus aureus* (Sa) and methicillin-resistant *S. aureus* (MRSA). Agar diffusion tests, sonication, turbidimetry, crystal violet staining, scanning electron microscopy and cell viability assays (fibroblasts, mesothelial cells) were performed to evaluate the response of the gels. Both compounds—especially the RIF-loaded gel—exerted a biocidal effect against gram-positive bacteria, developing wide inhibition halos, precluding adhesion to the mesh surface, and hampering bacterial survival in culture. The antibiotic gel proved innocuous, while lower viability was found in cells exposed to the antiseptic (*p* < 0.05). Together with their fast, affordable, convenient processing and easy application, the results suggest the potential effectiveness of these drug-loaded CMC gels in providing meshes with an antibacterial coating exhibiting great biocide performance.

## 1. Introduction

Surgical implantation of prosthetic mesh materials to repair abdominal wall defects is one of the most common procedures in general surgery, accounting for about 20 million interventions performed annually worldwide [1]. These biomaterials aim to provide mechanical support and stimulate natural processes of tissue repair and regeneration. Implanted meshes promote a tension-free repair, reducing the risk of undesirable side effects such as chronic abdominal pain, wound dehiscence, or hernia recurrence; factors contributing great advantages for patients in comparison with non-mesh repair techniques or autoplastic surgical approaches [2]. From the wide variety of marketed devices available, polypropylene (PP) meshes are extensively used, representing one of the best choices to treat abdominal wall tissue defects [3].

Nevertheless, implantation of PP meshes is not exempt from risks, comparable to any other biomaterial. Among the different postsurgical complications, device-associated infection is a devastating condition provoking strong impact on both patients and the healthcare system. The incidence of mesh infection is influenced by the surgical technique, the implanted device, the presence of contaminating bacteria and patient risk conditions, among other factors. For instance, rates of mesh infection range between 0.3% and 8% in ventral hernia repair [4], while incisional hernia exhibits rates of up to 30% [5]. Moreover, risk of infection dramatically increases in complex cases, entailing great risk of suffering bacterial colonization, such as bowel resection, or incarcerated or strangulated hernia [6].

At the onset of infection, the presence of a mesh in the host tissue diminishes the phagocytic activity of immune cells against bacteria and induces the expression of defense mechanisms in the metabolically active microorganisms [7]. The inflammatory response and macrophage activation commonly triggered following mesh implantation is exacerbated by bacteria. Hence, the release of pro-inflammatory cytokines, cytolytic enzymes and reactive oxygen species is augmented [8]. These alterations increase the risk of hernia recurrence, which would require further surgical interventions, raising morbidity and mortality rates. Together with this, prolonged hospital stays, additional treatments and resources used to treat those patients raise the economic costs, having a negative impact on the healthcare system [9].

The potential severity of prosthetic mesh infection makes it crucial to preclude early bacterial colonization of the implant. In the clinical setting, preoperative prophylaxis via systemic administration of antibiotics is standard practice. Although this approach aims to reduce postoperative infection rates, there is little consensus concerning its efficiency in hernia repair, given the multifactorial condition of this pathology [10,11]. Likewise, the misuse and abuse of antibiotics over the past decades is directly linked to the development of drug- and multidrug-resistant bacterial strains, which is currently one of the major threats to global health [12]. On this basis, there is clearly a need to develop strategies to hamper the interaction between bacteria and the implant surface, avoiding adhesion and killing microorganisms without using excessive or prolonged doses of antibiotics. Coating of meshes with drug-loaded compounds is currently an appealing approach to provide a local barrier that is capable of preventing bacterial colonization of the implant.

In this study, we have developed different antibacterial gels with the aim to assess a practical and manageable strategy for the prophylactic coating of hernia repair PP mesh materials immediately prior to implantation. These gels, made of sodium carboxymethylcellulose (CMC), a biocompatible compound with extensive application as an excipient and drug carrier, were functionalized with two biocide agents: either the antiseptic, chlorhexidine (CHX) or the antibiotic, rifampicin (RIF). The meshes were coated by soaking in the formulated gels and their performance was tested in vitro against *Staphylococcus aureus* (Sa) and methicillin-resistant *S. aureus* (MRSA), two of the main bacteria responsible for about three-quarters of the device-related infections following hernia surgery [13].

## 2. Results and Discussion

### 2.1. Solubility of Antimicrobials in the CMC Gel

Given the myriad properties displayed by this cellullose-derived compound, CMC is considered a significant carrier for drug delivery and other biomedical applications [14], which is why CMC was selected for the formulation of antimicrobial gels. During the elaboration of the base gel at the established concentration (1% *w*/*v*), CMC exerted great affinity for water, being completely dissolved by stirring, with no evidence of compound aggregation. For the preparation of the antibacterial gels, the dose of drug loaded (0.05% CHX; 0.13 mg/mL RIF) was determined by previous titration of the antibacterial activity and cytocompatibility through agar diffusion and cell viability tests (ranges: 2–0.025% and 1–0.01 mg/mL for CHX and RIF, respectively). The antibiotic RIF, manufactured as a lyophilized powder, is water-insoluble, requiring an organic solvent such as dimethyl sulfoxide (DMSO). Once reconstituted, RIF was handily mixed in CMC, forming an orange-amber colored solution which remained stable over time. By comparison, the CHX-loaded gel required different steps to be prepared. This antiseptic is highly cationic and therefore it can turn incompatible with anionic compounds such as CMC [15], especially if the drug is used at too high a concentration. When attempting to dilute the commercial CHX solution (20% *v*/*v*) into the base gel, insoluble precipitates formed as a result of this combination. For this reason, the antiseptic was first diluted in water and the resulting solution was subsequently thickened by the addition of CMC powder. All the gels formulated with these approaches exhibited a clear, homogeneous appearance, showing no impurities or variations once stored. Likewise, a slight, similar consistency of the three gel-based solutions which remained unaltered was observed during the whole study.

### 2.2. Spectral and Morphological Characterization of the Gels

**Figure 1:**
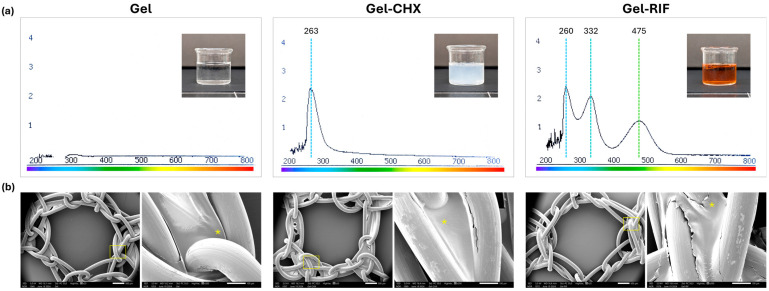
Spectral and morphological characterization of the gels. (**a**) UV-Vis absorption spectra of the formulated gels, with data of the maximum absorbance peaks recorded (nm) and macroscopic picture of freshly prepared gels. (**b**) Scanning electron microscopy (SEM) visualization of coated PP meshes with the corresponding Gel, Gel-CHX or Gel-RIF compounds (magnification ×25; scales 100 µm). Boxed areas were magnified (×200; scales 500 µm) for a better identification of the coating (*) accumulated in areas of filament knots.

Before being used as mesh-coating compounds, the formulated gels were characterized by UV-Vis spectral analysis (Figure 1a). As expected, no signal was detected in the base gel containing only CMC. The maximum peak for Gel-CHX (abs. 2.400) was recorded at 263 nm, in close correlation to data recently reported by others [16]. For Gel-RIF, different peaks were recorded at 260 (abs. 2.275), 332 (abs. 1.966) and 475 nm (abs. 1.125). Extrapolation of these maximum peak absorbances into calibration curves eased the quantification of the real concentration of the drug loaded in the gels. For Gel-CHX, the data collected revealed an average amount of 0.0487% CHX (y = 0.0248x − 0.0108; R^2^ = 0.8707). Concentration of RIF was quantified at the different peaks recorded, yielding values of 0.1923 mg/mL (y = 0.053x + 0.0717; R^2^ = 0.9059), 0.18593 mg/mL (y = 0.055x + 0.0778; R^2^ = 0.9495) and 0.17568 mg/mL (y = 0.0782x + 0.0877; R^2^ = 0.9556) at 260 nm 332 nm and 475 nm, respectively. The presence of multiple peaks in the UV-Vis spectra of RIF in aqueous solutions or even loaded into polysaccharide compounds has been previously reported in the literature. For instance, in a study involving acetylated amylose carrying RIF, two peaks were detected at 333 and 472 nm [17], which aligns with our experimental findings. In our outcomes, a third peak was also recorded at a lower wavelength (260 nm). This peak presumably corresponds to the DMSO used in the lyophilized antibiotic reconstitution, since this solvent shows a range of maximum absorbance at 260–280 nm depending on the concentration [18]. For each gel, three spectra were recorded using different compounds elaborated independently, which yielded equivalent outcomes and demonstrated the accuracy of this technique. Despite this, the spectral characterization of the gels should be combined with other precise analytical techniques such as Fourier transform infrared spectroscopy (FTIR) or rheological studies to provide a greater, more comprehensive characterization of the antibacterial coatings formulated in this study.

The morphological assessment of the gels used as coatings was performed by scanning electron microscopy (SEM) (Figure 1b). First, fragments of a bare PP mesh were visualized to characterize its structure, architecture and surface. This device displayed a reticular configuration produced by the knitting of smooth PP monofilaments with an average diameter of 162.0 ± 1.633 µm. The distribution of filaments created wide geometrically shaped pores with an effective area of 6.33 ± 0.042 mm^2^. Visualization of the coated meshes revealed an adequate association between the gels and the PP material. Filaments exhibited a thin and continuous layer of coating compound homogeneously covering their surface, which was only interrupted in the pore areas, where no traces of gel were evidenced. A greater accumulation of coating compound was located in the areas of the knots.

In a setting of mesh-related infection, surface roughness, the presence of filament clefts or even knots are potential niches that facilitate bacterial settlement [19]. For instance, recent experimental data show that hydrophobic devices exhibiting minimal changes in roughness can modulate up to 75-fold the amount of bacteria adhered to their surface [20]; a fact that makes roughness a key parameter for bacterial adhesion. Further, a greater amount of antibacterial coating in these zones will enhance its response in the most vulnerable regions of the implant.

### 2.3. Gel Flow Time

The gels were assessed to monitor flow time, in order to record any changes that could be attributed to the drug functionalization. At 4 °C, flow time recorded was very similar among the base Gel (17 s), Gel-CHX (19 s) and Gel-RIF (19 s). As expected by the known effect of temperature on a certain fluid density, a faster flow time was recorded for all groups at 23 °C (Gel 13 s; Gel-CHX 14 s; Gel-RIF 14 s) and 37 °C (Gel 12 s; Gel-CHX 13 s; Gel-RIF 12 s), although these differences were non-significant at any of the temperatures tested. Ultrapure water showed almost identical flow time during the whole study (11, 10, 9 s at 4 °C, 23 °C and 37 °C, respectively). The close similarities found between the data collected at 23 °C and 37 °C may suggest a consistent behavior of these gels at both room and body temperatures. As with many other compounds, viscosity of CMC-based solutions is related to the type of CMC, environmental temperature and concentration [21]. The CMC used for the elaboration of the antibacterial gels is described by the supplier as a medium viscosity formulation, which explains the relative speed of flow time recorded in this study. Given its properties, medium viscosity CMC is considered a great candidate for drug release and other biomedical applications [14]. In alignment with this, our experimental data reveal that the addition of an antiseptic or antibiotic does not alter or modify the viscosity of the base gel at the concentrations tested. In a theorethical clinical translation, a low-viscosity antibacterial gel may represent a beneficial feature to be used as prophylactic coating for medical devices in the surgical theatre, since the coating itself would not be causing any morphological variations or provoking impact during implantation of meshes.

### 2.4. Performance of Gels as Coating Compounds for PP Meshes

With the aim to determine the amount of coating retained on the surface of the PP mesh, fragments (1 cm^2^) were weighed before and after soaking in the corresponding gels. Consistent with the lightweight feature of this PP biomaterial [22], the uncoated meshes yielded an average weight of 5.85 ± 0.118 mg. Following soaking in the drug-free CMC gel, weight measurements revealed 16.03 ± 1.077 mg/cm^2^ of coating retained in the surface of the meshes. When antimicrobial gels were used, a significant increase in weight was recorded (*p* > 0.001), reaching values of around 80% for Gel-CHX (29.12 ± 1.544 mg/cm^2^) and 56% for Gel-RIF (25.98 ± 0.853 mg/cm^2^). It is reasonable that the loading of a drug entails an increase in weight of the carrier, based on the molecular weight of the drug itself. In this study, although non-significant, a higher weight was recorded for the antiseptic-loaded gel. According to the US National Library of Medicine (“PubChem” National Center for Biotechnology Information), molecular weights of CHX and RIF are 897.8 g/mol and 822.9 g/mol, respectively; data that align with our experimental results. A recent study involving hydrogels for drug release stressed the importance of balancing the molecular weight of both carrier and drug to optimize the performance of these systems [23]. This means that the balance between the molecular weight of the antimicrobial and the vehicle is a key feature for the design of improved antibacterial gel-based coatings that should not be overlooked.

One of the main goals of this study was to elaborate a compound that enables a straightforward coating of biomaterials exerting antibacterial activity. Given their water-based composition, the gels formulated here fulfilled this objective, allowing the soaking of meshes with no need to perform complex or additional steps. Notwithstanding the above, the aqueous nature of the gels does not provide strong bonding to the surface of the PP mesh, given the lack of functional groups and the low surface energy of this material [24]. This could be considered as a constraint for the long-term efficacy of the coating once the mesh is implanted in the host tissue. In this context, the incorporation of crosslinking agents and/or the modification of the surface charges of the mesh would enhance chemical bonding between the mesh and the gel, improving its in vivo performance.

### 2.5. Control of the Bacterial Inocula

The development of in vitro experiments with planktonic bacteria entails the risk of producing suspensions with a different number of microorganisms, thus augmenting the deviation among the data collected. Similarly, the existence of a 24 h time lag between the establishment of the inoculum and the determination of the number of viable bacteria by standard microbiological methods is of great relevance and may influence the reproducibility of the experiment [25]. One of the technical challenges arising from the assessment of antimicrobial susceptibility tests is the so-called “inoculum effect” [26], which entails the risk of under-/overestimating the required dose of drug, based on an inaccurate density of microorganisms in the testing inocula. In our study, every suspension prepared was routinely counted to monitor the number of viable bacteria, quantified as colony forming units (CFU) per unit of volume (mL). Elaboration of working inocula was performed following a standardized spectrophotometric protocol based on McFarland turbidity standards. For both strains, the number of bacteria was consistent in all the suspensions established (Table 1). The homogeneity among inocula demonstrates the reproducibility and replicability of this method, which is of great importance to evaluate the effect of biocides against bacteria [27].

### 2.6. Antibacterial and Biocide Performance of the Gel-Based Coatings

**Figure 2:**
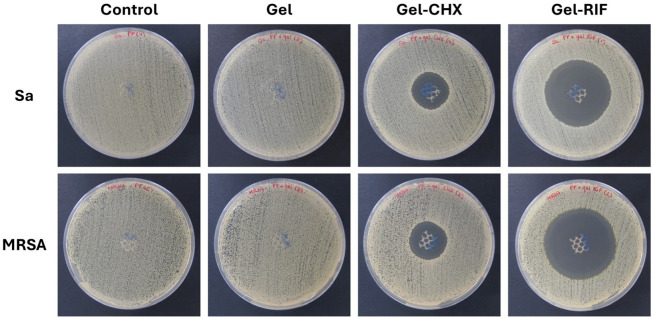
Agar well diffusion test. Macroscopic pictures of the *S. aureus*—(Sa) and MRSA-inoculated agar plates following 24 h of incubation with the PP meshes from the different groups. While control and Gel-coated meshes were fully colonized by bacteria, zones of inhibition (ZOI) were recorded in the agar plates containing antibacterial Gel-CHX and Gel-RIF meshes.

The agar well diffusion test was used to evaluate the antibacterial effect of the formulated gels (Figure 2 and Table 2). For both strains tested, zones of inhibition (ZOI) were recorded in all PP meshes that received antimicrobial coatings, with the Gel-RIF groups showing a significantly higher amplitude in comparison with the Gel-CHX ones (*p* < 0.01). The differences found between compounds can be attributed to the specific mechanisms of action of each drug. RIF is considered one of the most potent broad-spectrum antibiotics exerting a strong effect against gram-positive strains even when bacteria are found intracellularly. This antibiotic can permeate bacterial walls and crosses the membrane via passive diffusion. Inside the protoplasm, RIF provokes a fast inhibition of the bacterial RNA polymerase through its binding to the RNA polymerase β-subunit (RpoB). The resulting antibiotic–enzyme binding creates a stable complex that blocks the DNA-dependent RNA synthesis early, avoiding the elongation of the chain from the second or third nucleotide onwards. As a consequence of this, truncated RNA transcripts are not translated, and the protein synthesis is irreversibly inhibited [28,29]. CHX is one of the most widely used antiseptics given its potent biocidal activity, based on the destabilization of the bacterial membrane electric charges. This is a divalent cationic biguanide molecule, whose charges are attracted by the negatively charged surface of bacteria’ cell walls. The interaction between the antiseptic and the bacterial surface provokes an osmotic imbalance in the microorganism, triggering the flow out of K^+^ ions and the loss of metabolic energy, leading to a downstream of the cellular respiration process, membrane disruption and subsequent leakage [30,31]. While RIF has been reported to exert bactericidal effect against staphylococcal strains even at very low concentrations [32], CHX can be bacteriostatic if the dose is insufficient, or if the antiseptic is rapidly removed or diffused out of the site of action [33]. In vitro, the superiority of bactericidal drugs over the bacteriostatic ones seems to be clear and it would not even be uncommon to believe that the former is preferable to the latter in eradicating gram-positive bacteria. However, it should be noted that these differences are not so relevant for a potential clinical application, as there are many other key factors to be considered, such as pharmacokinetics, pharmacodynamics and the tissue/fluid penetration parameters of the drug [34].

While the antiseptic-loaded gel developed halos with similar ZOI between Sa and MRSA, the antibiotic coating exerted a more intense activity against MRSA (*p* < 0.01). Ordinarily, it could be conceivable that a strain exerting resistance against a certain antibiotic (i.e., methicillin) would be more difficult to treat with other drugs than its sensitive counterparts. In the specific case of Sa and MRSA, it has been reported that different antibacterial drugs such as many antibiotics [35], antiseptics [36] or even natural mycotoxins [37] exert a higher effect against the latter, which is in agreement with our observations and highlights the importance of testing antimicrobials with different bacterial strains or isolates.

**Figure 3:**
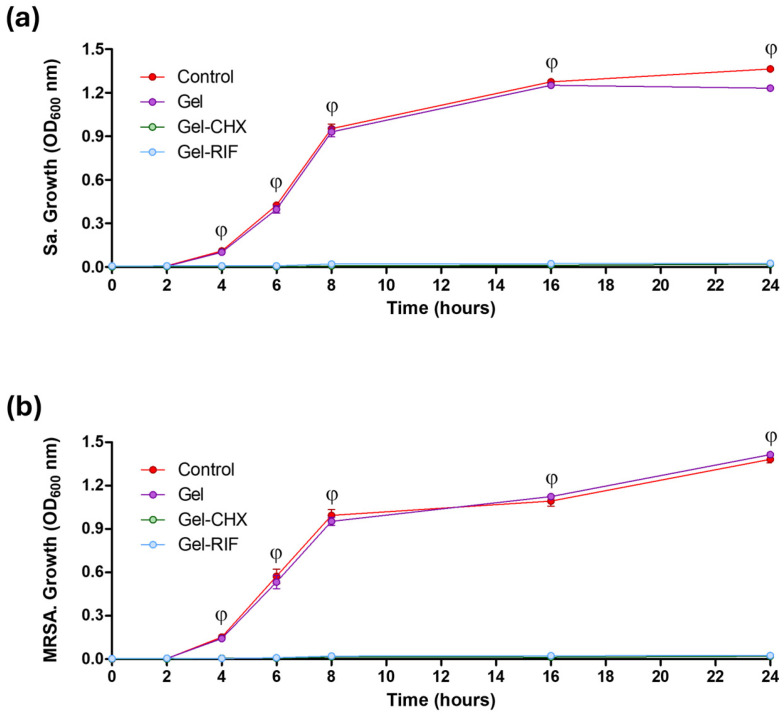
Inhibition of bacterial growth. Graphs represent the growth curves of (**a**) *S.aureus* (Sa) and (**b**) MRSA influenced by the presence of the different formulated gels in a 24 h period of study. Measurement of bacterial growth was carried out using a spectrophotometric protocol. For both strains, similar growth was recorded in groups receiving control and Gel meshes, while no growth was monitored in any culture exposed to the antimicrobial gels. Statistical evaluation of the growth curves: φ: both Gel-CHX and Gel-RIF vs. both control and Gel groups (*p* < 0.001).

Along with the potential to prevent adhesion, the biocidal activity of the formulated gels was also monitored. When Sa and MRSA were cultured in the presence of either Gel-CHX or Gel-RIF coated meshes, their growth pattern was inhibited, as observed by turbidimetric assays (Figure 3). This effect was not exerted by the unloaded gel, which allowed an exponential bacterial growth equivalent to that of the control cultures, turning statistically significant compared to the antibacterial-loaded groups after 4 h of culture (*p* < 0.001). The ability to inhibit bacterial growth can be considered as an advantageous feature for device coatings. In hernia repair, it has been established that the presence of an implanted biomaterial significantly reduces the minimum dose of bacteria capable of developing an infection to as low as 10^2^ CFU [38]. Given that it takes about 30 min for staphylococcal strains to replicate, a single bacteria would trigger a device-related infection in less than 4 h postoperatively. Therefore, if a coated device avoids early bacterial growth once implanted, its antimicrobial effectiveness will be enhanced. In this regard, there are other literature reports describing encouraging outcomes on the prophylactic soaking of devices and prostheses in antimicrobial solutions. Such is the case of vancomycin and gentamycin, which are used to irrigate tissue substitutes in ligament reconstruction [39]; or the antiseptic bacitranin for the prophylactic immersion of surgical sutures [40]. These and other data bring into light the relevance of providing the device with an antibiotic- or antiseptic-based antibacterial barrier that would inhibit bacterial growth, or even destroy them, hampering the onset of infection.

### 2.7. Usefulness of the Gel Coatings to Prevent Bacterial Adhesion to the Mesh Surface

**Figure 4:**
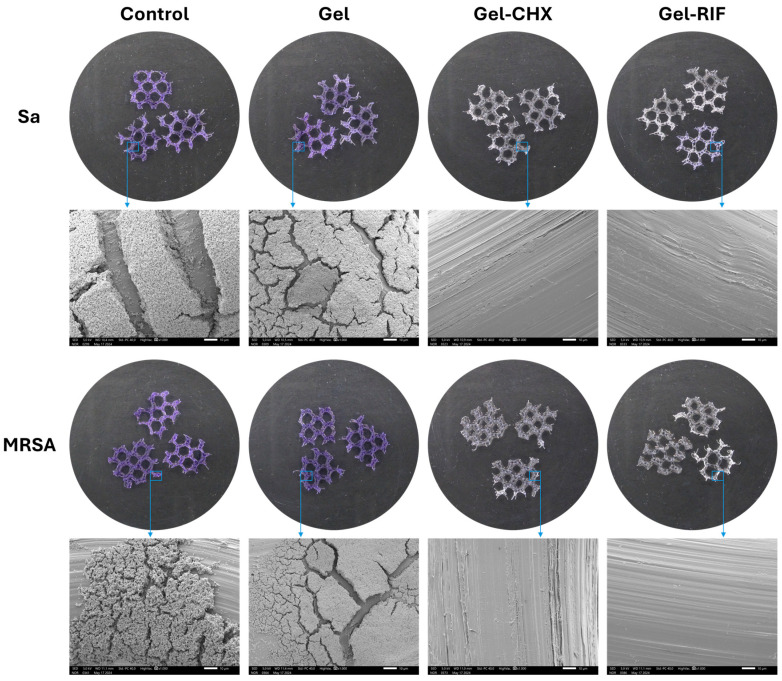
Bacterial colonization of the mesh surface. The strong bacterial biomass adhered to the surface of the control and Gel-coated meshes was observed by crystal violet staining, which provided an intense purple tone in those areas of the mesh with bacteria adhered (macroscopic pictures) and subsequently confirmed under SEM visualization, where thick colonies of microorganisms were observed to be strongly adhered to the mesh filaments (magnification ×1000; scales 10 µm). By contrast, both antibacterial gels exerted biocidal activity that killed bacteria and subsequently hampered adhesion of *S. aureus* (Sa) and MRSA to the PP filaments.

With the aim to evaluate the potential of the drug-loaded gels to prevent adhesion of Sa and MRSA to the surface of meshes, different visualization and quantitative methods were performed. Macroscopic and microscopic observations of the different groups (Figure 4) evidenced that both antibacterial gels avoided adhesion of Sa and MRSA to the surface of the coated PP meshes. Likewise, crystal violet staining revealed no bacterial biomass on the meshes coated with either Gel-CHX or Gel-RIF, in contrast to the control and the Gel groups, whose surfaces were completely stained, suggesting a great presence of bacteria in those samples. These observations were validated at higher magnification under SEM, where strong adhesion of Sa and MRSA was visualized in both control and Gel groups, while PP filaments from meshes coated with Gel-CHX and Gel-RIF displayed a surface free of bacteria. It should be noticed that, although these coatings exert optimal biocide activity, they are not repelling bacterial attachment. The lack of persistent bacteria in the surface of meshes is suggestive of the fast speed of these drug-loaded coatings to kill Sa and MRSA before they would adhere to the mesh filaments, which could be indirectly related to an antiadhesive or antifouling performance of the gels.

The macroscopic and microscopic findings described were corroborated by sonication (Table 3). Quantification of adhesion yielded high loads of living bacteria in those meshes not protected by the action of antibacterial gels, especially when materials were challenged with Sa (*p* < 0.01). Contrary to this, no CFU were collected either from the Gel-CHX or the Gel-RIF groups, suggesting the absence of viable bacteria adhered to the surface of these meshes due to the rapid biocide effect of the drugs.

Taken together, the macroscopic, microscopic and quantitative findings demonstrate the high performance of these biocidal gels to prevent colonization and survival of bacteria adhered to the surface of PP meshes for hernia repair. One of the most crucial requisites that an antimicrobial biomedical device should fulfil to properly fight against device-related infection is to preclude bacterial adhesion. The interaction of bacteria with the prosthetic surface and subsequent adhesion represents the initial step in the pathway for infection [41]. Once in contact with the biomaterial, bacteria undergo a fast, reversible physicochemical association with its surface, followed by an adhesin protein-based attachment which turns irreversible [42]. Bacterial colonization of an implant can evolve towards a biofilm infection when adhered microorganisms start to produce exopolysaccharide matrix. This matrix acts as a protective capsule which preserves bacteria from the action of drugs and host immune cells [43]. Infections involving biofilms can be devastating, since many of the antibiotics available fail to eradicate bacteria in such a scenario. In many cases, the lack of effective treatment entails the need to perform removal of the prosthesis and debridement of the area before proceeding with reimplantation, with the obvious consequences for both the patient and the healthcare system.

There is preclinical and clinical evidence that soaking or dipping meshes in antimicrobial solutions prior to implantation is an adequate strategy to reduce the risk of developing postoperative infections [44,45]. In previous work, we demonstrated the ability of CHX and RIF to act against a preformed mesh-associated biofilm when the drugs were loaded in a thermo-responsive hydrogel [46]. In this study, our findings suggest a potential capacity of these antimicrobials to influence the bacteria-surface interaction and jeopardize adhesion before it becomes irreversible, meaning that the prophylactic use of these gels with meshes could help preclude the early colonization of bacteria. In the experimental model developed, a 10^6^ CFU dose of bacteria was used to inoculate the samples. This is considered a high dose, which is about four loads higher than the minimum number of bacteria required to establish infection in a medical device [38]. Likewise, under normal conditions only a low number of bacteria enter the wound, where they start growing and colonizing the mesh [38]. By reproducing an acute infection such as this, it is possible to ascertain whether the biocide activity of these coatings is enough to control the infection in the biomaterials tested. Due to this and based on our findings, we hypothesize that the antibacterial Gel-CHX and Gel-RIF coatings would kill bacteria which had eventually arrived at the surgical area, making these great candidates for a prophylactic coating of meshes.

### 2.8. Cytocompatibility

Apart from the activity exerted by the bioactive agent chosen, one of the key features determining the performance of an antibacterial coating is the vehicle in which the drug is loaded. An optimal coating for hernia repair mesh materials must be non-toxic and degradable. Likewise, coating compounds cannot alter the biomechanical response or tissue integration into the host tissue [47]. Ideally, it should also exert controlled drug release over time if polymer compounds are used. In this study, CMC was selected as the vehicle for the development of the antibacterial gel. This is a cellulose-based polymer with proven biocompatibility, high shape fidelity, stability and gel-forming ability, frequently utilized in tissue engineering, wound dressing and drug carriers, and successfully tested for the sustained release of drugs in hydrogels [48].

**Figure 5:**
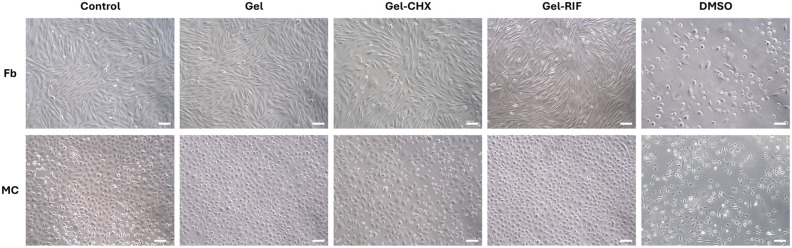
Cell viability. The figure shows representative micrographs of fibroblasts (Fb) and mesothelial cells (MC) following 24 h of exposure to the different gels and dimethyl sulfoxide (DMSO) as a positive control for cell toxicity (magnification ×100; scales, 100 µm). In all cases, the gels exerted no detrimental effect on cells, whose morphology, shape and proliferative status was identical to that observed in the control cultures.

Gels formulated with this compound exerted null detrimental effect on fibroblast (Fb) and mesothelial cell (MC) cultures, evidencing the safety of CMC for this application. When the gel was functionalized with the drugs, a different response was observed depending on the type of the antimicrobial used (Figure 5 and Table 4). While the antibiotic was fully innocuous, a slightly toxic effect was recorded for CHX over both Fb and MC (*p* < 0.05) as percentages for cell viability were recorded around 70%; a value considered as a threshold for toxicity according to ISO 10993–5 in vitro biocompatibility testing of medical devices guidelines [49]. In alignment with this, cultures exposed to the antiseptic displayed mild morphological changes and lesser mitotic figures compared to the rest of the groups. The toxicity of CHX towards eukaryotic cells is well known, being attributed to its intrinsic mechanism of action, and has been concisely reported in the past [50]. Nevertheless, it is considered one of the best antiseptics to be used in biomedical applications given its powerful effect and prolonged action over time [51]. In the specific field of hernia repair, this antiseptic has been used in the manufacture of the first antibacterial mesh with US Food and Drug Administration (FDA) approval [52], bringing to light the usefulness of CHX for the development of efficient antibacterial devices. Similarly, RIF has also been cleared by the FDA to produce an antibiotic-releasing collagen-based device for soft tissue reinforcement [53].

### 2.9. Limitations and Future Perspectives on the Use of Gel-Based Coatings for Devices

During the surgical repair of defects involving soft tissues, such as hernia, it is not uncommon for surgeons to immerse surgical sutures and prosthetic devices in antimicrobial solutions as a preoperative strategy to prevent settlement of bacteria along the implant [44,45]. Particularly noteworthy is the fact that applying a local prophylaxis to meshes may help reduce the extensive systemic administration of antibiotics, considered one of the main healthcare concerns given the growing proliferation of bacterial resistance to drugs. Although this is an appealing approach, it has not yet been established as a routine procedure during the surgical act, and more research on this basis is being requested by clinicians, to determine its applicability in patients [54].

In this study, we have evaluated the performance of two antibacterial gels developed for the coating of PP meshes typically used in the surgical repair of abdominal hernia pathology. Based on the outcomes recorded, both gels (especially the antibiotic-loaded one) exerted great activity against Sa and MRSA. As previously mentioned, these strains are among the main bacteria responsible for provoking postoperative biomaterial-related infections, affecting not only mesh materials but also the wide variety of implantable devices for clinical use available [55]. Given its strong impact, any technological approach aimed at reducing the risks of developing an infection would entail great benefit for both society and healthcare systems. The gels were elaborated using a straightforward method, with the purpose of easing its application on the meshes immediately prior to implantation. If an eventual clinical translation is possible, this strategy would be applied in situ with virtually all biomaterials, regardless of their structure, shape, material composition or intended use. The versatility of the compound used as a vehicle may also facilitate the gel functionalization with other drugs, alone or combined, as a convenient, tunable prophylactic system. For instance, these gels could be manufactured and provided to hospitals as a sterile, ready-to-use coating compound to be used in theatre. To our knowledge, there is a great scarcity of products such as this, making the idea of using these coatings an innovative and accessible way to fight against postoperative infections.

Although the findings described in this study are encouraging, it must be stressed that outcomes were collected under in vitro conditions, providing limited information and therefore the response of these compounds needs to be examined further. A more in-depth physicochemical assessment and relevant data regarding safety, dosage, stability over time, degradability, effectiveness against gram-negative strains and/or clinical isolates, other drug candidates, or applicability with other devices apart from PP meshes are still missing. In this context, running preclinical validation and clinical trials would provide a more comprehensive overview of the potential applicability of these antimicrobial gels and standardize its use with biomaterials as a new tool to fight against bacterial infection.

## 3. Conclusions

The drug-loaded CMC gels formulated in this study displayed a convenient elaboration and easy application for endowing biomaterials with antibacterial coating, encouraging their potential use with most implantable devices, regardless of their shape, structure or chemical composition. The biocidal activity of the formulated compounds was optimal against gram-positive bacteria such as *Staphylococcus aureus* and MRSA; the strains responsible for most of the device-related infections. Moreover, the antibiotic-loaded gel especially evidenced strong cell compatibility, which could facilitate its local application, entailing low risk of detrimental effects for host cells. Its performance as a prophylactic coating for PP meshes subtly suggests a potential use of these antibacterial gels in an attempt to reduce the rate of postoperative device-related infection following hernia repair.

## 4. Materials and Methods

### 4.1. Chemicals

To prepare the gels, the following chemicals were purchased. Medium-viscosity sodium carboxymethylcellulose (formula (C_12_H_14_O_9_R_6_)_n_; reference C4888; CAS number 9004-32-4; density 400–800 cP 2% in H_2_O, 25 °C; degree of substitution 0.65–0.90 carboxymethyl group per anhydroglucose unit), rifampicin (formula C_43_H_58_N_4_O_12_; reference R3501; CAS number 13292-46-1; molecular weight 823.0 u.; purity ≥ 97%) and dimethyl sulfoxide (formula (CH_3_)_2_SO; reference D8418; CAS number 67-68-5; molecular weight 78.13 u.; purity ≥ 99.9%) were provided by Sigma-Aldrich (St. Louis, MO, USA). A 20% *v*/*v* chlorhexidine digluconate solution (formula C_22_H_30_Cl_2_N_10_.2C_6_H_12_O_7_; reference sc-252570; CAS number 18472-51-0; molecular weight 897.76 u.; density 1.06 g/cm^3^, in H_2_O, 20 °C) was provided by Santa Cruz Biotechnology (Santa Cruz, CA, USA).

### 4.2. Elaboration of the Gels

The chemicals utilized to elaborate these gels were used as received. First, a base gel was developed under sterile conditions using sodium carboxymethylcellulose (CMC) and two bioactive antibacterial gels were formulated by loading into the base gel the antiseptic chlorhexidine (CHX) or the antibiotic rifampicin (RIF). For the elaboration of the unloaded gel, a solution of 1% *w*/*v* CMC in sterile ultrapure water was prepared and gently stirred at room temperature until completely dissolved. Both bioactive gels were prepared according to the manufacturer’s recommendations for drug reconstitution. For the antiseptic-loaded gel, a dilution of 0.05% *v*/*v* CHX in sterile ultrapure water was prepared and subsequently 1% *w*/*v* CMC was added, with continuous stirring at room temperature. The antibiotic RIF was first reconstituted in dimethyl sulfoxide (DMSO) and mixed with 1% CMC to prepare a 0.13 mg/mL RIF-loaded gel solution. All gels were protected from light and stored at 4 °C until use.

### 4.3. Spectral Characterization of the Formulated Gels and Determination of Drug Content

A UV-Vis characterization of the different gels was performed using spectrophotometric assays. Once prepared, the drug-free Gel, the Gel-CHX and the Gel-RIF compounds were allowed to stabilize for 24 h at room temperature, and antimicrobial-loaded gels were then diluted 1:3 in CMC base gel to avoid surpassing the detection threshold of this method. The UV-Vis absorption spectra of the 3 gels were recorded in triplicate, using an Ultrospec 3100 Pro spectrophotometer equipped with a 10 mm matched quartz cell (Amersham Biosciences, Little Chalfont, UK), establishing a spectral range of 200–800 nm and a wavelength accuracy of 1 nm. To quantitatively determine the amount of drug loaded in the gels, absorbance corresponding to the peak-wavelength recorded in these spectra for CHX (263 nm) and RIF (260/332/473 nm) was extrapolated to calibration curves elaborated using series of known concentrations (ranges: CHX 0.0125–0.075% and RIF 0.10–0.25 mg/mL). For each curve, concentrations tested were diluted 1:3 to keep the same experimental conditions used in the spectral characterization.

### 4.4. Monitoring of the Flow Time

To determine whether the loading of CHX or RIF affects the viscosity of the antibacterial gels, an assay was conducted to monitor the flow time for these compounds. The test was based on ISO 2431:2011 “Determination of flow time by use of flow cups” with brief modifications [56]. For this purpose, a Ford viscosity cup with 4 mm orifice diameter and 100 mL capacity was used. With the cup fixed on a platform and the orifice blocked, the cup was filled to its maximum with the corresponding gel and the orifice was subsequently opened to let the compound fluid out of the cup. To determine the flow time, the elapsed time (s) between the onset of the flow and the moment when the flow stream first breaks off close to the orifice was monitored. For this experiment, the gels were prepared under the conditions described and allowed to stand refrigerated for 24 h. For each gel, flow time was monitored at 4 °C, 23 °C and 37 °C, and sterile ultrapure water was used as control.

### 4.5. Mesh Material

The biomaterial Optilene Mesh Elastic (B. Braun, Melsungen, Germany) was selected. This is a reticular, lightweight, monofilament PP mesh for the surgical repair of abdominal wall defects. Squares with dimensions of 1 cm^2^ of the PP mesh were cut and sterilized in a Sterlink Mini low-temperature plasma sterilizer (Plasmapp Co., Ltd., Seoul, Republic of Korea).

### 4.6. Coating the of Meshes and Establishment of the Study Groups

The meshes were coated by means of the soaking method without using any crosslinking or adhesive agents that would stimulate bonding of the gels to the PP filaments. Briefly, the samples were immersed in vials containing 1 mL of the corresponding gel for 5 min at room temperature and handled with sterile tweezers and meshes were used in the different experiments immediately after coating. Before transferring the samples to the corresponding solid agar or liquid broth media, the coatings were allowed to drop off the excess of liquid to ensure that only the thin film of coating impregnating the PP filaments was carried. According to the coating received, the following study groups were established:

- Control: Uncoated PP meshes.
- Gel: PP meshes coated with unloaded gel.
- Gel-CHX: PP meshes coated with 0.05% CHX-loaded gel.
- Gel-RIF: PP meshes coated with 0.13 mg/mL RIF-loaded gel.

### 4.7. Surface Characterization of the Coated Meshes

The amount of gel retained to the surface of the meshes and the homogeneity of the coating were determined by quantitative and visual methods. First, uncoated fragments of the PP mesh were weighted using a Mettler AE240 dual range balance (Mettler Toledo, Columbus, OH, USA). Then, these fragments were randomly soaked in the gels (n = 6 each) as previously described and weighted again to record the quantity of coating per cm^2^ of surface. Following these measurements, coated meshes were air-dried overnight in an AV 30/70 class-IIa laminar flow cabinet (Azbil Telstar SLU, Barcelona, Spain). Dried samples were placed onto aluminum pins, gold-palladium sputtered and visualized using a JSM-IT500 InTouchScope™ scanning electron microscope (SEM) (Jeol Ltd., Tokyo, Japan) set with the following parameters: high vacuum, SED 5 kV; working distance 16 ± 1 mm; Std.-PC 50.0; scanning time 40 seg, image resolution 2560 × 2048 ppp. Uncoated meshes were included as a control.

### 4.8. Bacterial Strains

To determine the antibacterial performance of the gel-based coatings, two staphylococcal strains were used, Sa ATCC25923 and MRSA ATCC43300 (Spanish Collection of Type Cultures, CECT, Valencia, Spain). To avoid cross-contamination between strains, all the experiments were performed in an independent manner. For each bacteria, 1–2 fresh colonies were picked from a lysogeny broth (LB) agar plate (BioMerieux, Marcy-l’Étoile, France), transferred to 10 mL of fresh LB broth and incubated at 37 °C for 3 h. Then, absorbance was read by spectrophotometry (wavelength 600 nm) and adjusted in sterile 0.9% saline until values equivalent to a 0.5 McFarland suspension (0.340–0.360 nm) were reached. Next, a 100-fold dilution in sterile 0.9% saline was performed to establish the working inoculum at a concentration of 10^6^ CFU/mL. The number of viable bacteria in every inoculum prepared was determined according to standard microbiological procedures of colony counts.

### 4.9. Agar Diffusion Test

The antibacterial effect of the formulated gel-based coatings was assessed by means of the agar well diffusion method. For each of the strains, bacterial lawns were spread in 20 LB agar plates by inoculating 0.1 mL of the 10^6^ CFU/mL suspension on the surface of the agar and subsequent spreading with sterile cotton swabs. Then, the meshes were soaked in the corresponding gel (n = 5 per group per strain) as previously described, and individually placed on the surface of the inoculated agar. Plates were incubated for 24 h at 37 °C to promote the development of zones of inhibition (ZOI). Following incubation, plates were photographed and the ZOI amplitude was quantified by measuring two perpendicular diameters using the open-source software for image analysis Fiji 2.14 (https://fiji.sc/; accessed on 12 May 2024).

### 4.10. Turbidimetry

The potential of the formulated gels to inhibit bacterial growth in culture was assessed by a turbidimetric approach. For each strain, 1 mL of suspension at the working concentration (10^6^ CFU/mL) was inoculated in 3 mL of LB broth using P-6 plates. Next, the meshes were coated, individually transferred into the wells (n = 5 per group per strain) and incubated for 24 h at 37 °C. During incubation, at the selected time-points (0, 2, 4, 6, 8, 16, 24 h), 100 µL aliquots were collected for each well and absorbance was read (600 nm) using an iMark microplate absorbance reader (Bio-Rad Laboratories Inc., Hercules, CA, USA) to monitor bacterial growth over time upon exposure to the different gel coatings.

### 4.11. Sonication

To determine the ability of the gel coatings to prevent bacterial adhesion to the mesh surface, a sonication assay was performed., For each strain, 1 mL of suspension at the working concentration (10^6^ CFU/mL) was inoculated in 3 mL of LB broth using P-6 plates. Next, the meshes were coated, individually transferred into the wells (n = 5 per group per strain) and incubated for 24 h at 37 °C. Following incubation, the meshes were gently washed with 1 mL of 0.9% saline to remove non-adhered bacteria and immersed in sterile tubes containing 10 mL of 0.9% saline. Tubes were submitted to sonication at 40 KHz for 10 min using a Bransonic 3800-CPXH ultrasound bath (Branson Ultrasonics, Danbury, CT, USA) and vortexed for 1 min. Next, the supernatant was used to prepare 5 serial 10-fold dilutions in sterile 0.9% saline, plated in LB agar plates and incubated for 24 h at 37 °C for subsequent colony counts and quantification of the average number of bacteria adhered to the mesh surface per study group.

### 4.12. Crystal Violet Staining

As a complement of the quantification brought by the sonication procedure previously mentioned, crystal violet staining was carried out. This dye has a great affinity for peptidoglycans, staining the cell wall of gram-positive bacteria with a purplish tone, which allows for a macroscopic visualization of the biomass adhered to the surface of the meshes in the different study groups. For each strain, 1 mL of suspension at the working concentration (10^6^ CFU/mL) was inoculated in 3 mL of LB broth using P-6 plates. Next, the meshes were coated, individually transferred into the wells (n = 3 per group per strain) and incubated for 24 h at 37 °C. Following incubation, the meshes were fixed in iced 70% ethanol at −20 °C for 30 min, washed in sterile ultrapure water and stained with 0.25% crystal violet (Sigma-Aldrich, St. Louis, MO, USA) for 10 min. The stained meshes were gently washed with ultrapure water to remove the excess of dye and photographed.

### 4.13. Scanning Electron Microscopy (SEM)

With the aim to visualize the mesh filaments at high magnification and evaluate the performance of the gel coatings to hamper adhesion of bacteria to the mesh surface, SEM was used. For each strain, 1 mL of suspension at the working concentration (10^6^ CFU/mL) was inoculated in 3 mL of LB broth using P-6 plates. Next, the meshes were coated, individually transferred into the wells (n = 3 per group per strain) and incubated for 24 h at 37 °C. Following incubation, the meshes were fixed in 3% glutaraldehyde at 4 °C for 2 h, washed in sterile ultrapure water and dehydrated using an ethanol graded series (70%, 90%, 100%, 5 min-incubation each). Samples were placed onto aluminum pins, gold-palladium sputtered and visualized under SEM, using the same parameters previously detailed.

### 4.14. Cell Viability

To evaluate the safety of the formulated gels to eukaryotic cells, a cell viability test was performed using fibroblasts (Fb) and mesothelial cells (MC) of 3 healthy male New Zealand rabbits. Experimental animals belonged to another study in which our group is involved, with approval from the Committee on the Ethics of Animal Experiments of the University of Alcalá, Madrid, Spain (PROEX 047.7/22). Both Fb and MC were previously isolated from dermis and omentum tissue biopsies, respectively, following our standard protocols for cell harvesting and culture [57].

Cells were cultured in 25 cm^2^ flasks containing 3 mL of Dulbecco’s modified Eagle medium (DMEM) provided with 10% fetal bovine serum (FBS) and 1% pen-strep antibacterial mixture (all from Gibco/Life Technologies Corporation, Carlsbad, CA, USA). Flasks were cultured under controlled humid atmosphere (37 °C, 5% CO_2_) and visualized using a Zeiss Axiovert 40C phase-contrast inverted microscope (Carl Zeiss, Oberkochen, Germany). Media was renewed every 72 h.

Cells from the 2nd and 3rd passages were used for the viability assays. Both Fb and MC were seeded in 6-well plates at concentration 2.5 × 10^5^ cells/well and incubated overnight under controlled atmosphere. Next, 100 µL of the different gels were added to the wells (n = 5 per group), including a positive control for toxicity using 10% DMSO. Following a 24 h incubation, culture media was removed, cells were washed and FBS-free DMEM containing 10% alamarBlue colorimetric reagent (Bio-Rad Laboratories Inc.) was added into each well. Following a 5 h incubation period, several 100 µL aliquots were collected from each well and absorbance was read using a microplate absorbance reader (570 and 600 nm). To calculate the percentage cell viability of the different study groups, data were analyzed using the software tool provided online by the manufacturer (https://www.bio-rad-antibodies.com/colorimetric-calculator-fluorometric-alamarblue.html; accessed on 21 May 2024).

### 4.15. Statistical Analysis

All data collected were provided as mean ± standard error of the mean, being compared among the different study groups using the Mann–Whitney U test and one-way analysis of variance (ANOVA) with a post hoc Bonferroni test. Statistical tests were carried out using the GraphPad Prism 5.0 software (GraphPad Software Inc., La Jolla, CA, USA) for Windows with a level of significance set at *p* < 0.05.

## Figures and Tables

**Table 1:** Quantification of the viable bacteria (CFU/mL) found in the different inocula established.

Inoculum	*S. aureus* (Sa)	MRSA
#1	2.13 × 10^6^	2.13 × 10^6^
#2	1.51 × 10^6^	1.88 × 10^6^
#3	2.17 × 10^6^	2.03 × 10^6^
#4	1.79 × 10^6^	1.98 × 10^6^
#5	1.94 × 10^6^	2.01 × 10^6^

**Table 2:** Mean diameter of ZOI (mm) developed by the antibacterial gel-coated meshes.

Bacterial Strain	PP	Gel	Gel-CHX	Gel-RIF
*S. aureus* (Sa)	0	0	23.33 ± 0.51 ^(2)^	41.78 ± 0.24 ^(1)^
MRSA	0	0	24.54 ± 0.09 ^(3)^	43.49 ± 0.31

^(1)^ vs. Gel-RIF MRSA (*p* < 0.01); ^(2)^ vs. Gel-RIF Sa (*p* < 0.01); ^(3)^ vs. Gel-RIF MRSA (*p* < 0.01).

**Table 3:** Quantification of viable bacteria (CFU) yielded from the sonicated PP meshes.

Bacterial Strain	Value	Control ^(1)(3)^	Gel ^(2)(3)^	Gel-CHX	Gel-RIF
*S. aureus* (Sa)	Minimum	1.120 × 10^7^	3.420 × 10^6^	0	0
	Maximum	1.680 × 10^8^	1.620 × 10^7^	0	0
	Mean	4.306 × 10^7^	9.180 × 10^6^	0	0
	SD	6.985 × 10^7^	5.023 × 10^6^	0	0
	Error of mean	3.124 × 10^7^	2.246 × 10^6^	0	0
MRSA	Minimum	1.700 × 10^5^	1.610 × 10^5^	0	0
	Maximum	9.170 × 10^5^	3.170 × 10^5^	0	0
	Mean	4.274 × 10^5^	2.184 × 10^5^	0	0
	SD	2.878 × 10^5^	5.967 × 10^4^	0	0
	Error of mean	1.287 × 10^5^	2.669 × 10^4^	0	0

^(1)^ Sa vs. MRSA (*p* < 0.01); ^(2)^ Sa vs. MRSA (*p* < 0.01); ^(3)^ vs. antimicrobial gels Sa/MRSA (*p* < 0.001).

**Table 4:** Quantification of the cell viability (%) recorded by alamarBlue assay.

Cell Type	PP	Gel	Gel-CHX	Gel-RIF	DMSO
Fb	96.44 ± 1.38 ^(1)^	92.49 ± 2.40 ^(1)^	72.55 ± 1.27 ^(2)^	91.04 ± 2.79	19.17 ± 2.97 ^(3)^
MC	96.80 ± 1.37 ^(1)^	94.95 ± 3.92 ^(1)^	66.53 ± 1.14 ^(2)^	90.42 ± 0.79	17.63 ± 3.50 ^(3)^

^(1)^ vs. Gel-CHX (*p* < 0.001); ^(2)^ vs. Gel-RIF (*p* < 0.05); ^(3)^ vs. all groups (*p* < 0.001).

## Data Availability

The original contributions presented in the study are included in the article, further inquiries can be directed to the corresponding author.
